# Insight into early-phase trials for lung cancer in the United States

**DOI:** 10.1186/s40880-015-0027-5

**Published:** 2015-07-10

**Authors:** Jin-Ji Yang, Yi-Long Wu

**Affiliations:** Guangdong Lung Cancer Institute (GLCI), Guangdong General Hospital (GGH), Guangdong Academy of Medical Sciences (GAMS), Guangzhou, Guangdong 510080 P.R. China

**Keywords:** Early-phase trial, Lung cancer, Phase 1, Preclinical, Oncology

## Abstract

**Introduction:**

Few data have been published comparing early-phase trials for lung cancer between China and the United States (US). This study was to investigate the differences of phase 1 trials for lung cancer between these two countries.

**Methods:**

In 2014, a cross-sectional survey was conducted to compare phase 1 trials for lung cancer between the Guangdong Lung Cancer Institute (GLCI), the University of Wisconsin Carbone Cancer Center (UWCCC), and the University of Texas MD Anderson Cancer Center (MDACC).

**Results:**

We found that the GLCI had a lower percentage of phase 1 lung cancer trials than the MDACC in December 2014 (23.8% [5/21] vs. 59.8% [28/47], *P* = 0.006) and the UWCCC in September 2014 (16.7% [3/18] vs. 34.8% [8/23], *P* = 0.345). Descriptive analyses were performed for early-phase trials conducted by the Cancer Therapy Evaluation Program at the National Cancer Institute (CTEP/NCI), the MDACC, and the Chinese Thoracic Oncology Group (CTONG). There were 149 ongoing early-phase trials in the Department of Investigational Cancer Therapeutics (Phase 1 program) at the MDACC in October 2014. In contrast, no phase 1 trials had been initiated by the CTONG since its establishment in 2007.

**Conclusions:**

These data suggest that a significantly higher percentage of phase 1 trials for lung cancer were conducted in the US than in China. Early-phase oncology trials with robust preclinical data had a higher chance of being approved by the Investigational Drug Branch at the CTEP/NCI. Given the importance of early-phase oncology trials in developing innovative cancer medicines, such studies should be highly encouraged and strategically funded in China.

Non-small cell lung cancer (NSCLC) accounts for approximately 80% of primary lung cancer, which is the leading cause of cancer-related death in both men and women worldwide. More than one half (55%) of NSCLC patients with metastatic disease are medically and surgically incurable [1], leading to a poor prognosis with a median survival of 8–9 months [2, 3]. Therefore, more and more clinical trials have been designed to treat patients with advanced NSCLC. The Iressa NSCLC Trial Evaluating Response and Survival Versus Taxotere (INTEREST) trial [4], a global, multi-center, randomized, phase 3 trial conducted in 2004, was the starting point at which Chinese investigators contributed to making lung cancer history. Over the past decade, Chinese investigators have completed several phase 3 trials [5–8] that have greatly changed the clinical practice of treating lung cancer [9], including the Iressa Pan-Asia Study (IPASS), which was a milestone trial [5]. The Chinese Thoracic Oncology Group (CTONG) has launched more than 30 trials since its establishment in 2007. However, most of the ongoing trials for lung cancer at the CTONG are observational phase 2 and 3 studies [10]. Since its inception, the CTONG has yet to conduct any early-phase trials for lung cancer, particularly phase 1 trials.

Phase 1 studies of novel anticancer agents are a crucial step in antitumor drug development, as they translate years of bench studies to the bedside. The primary goal of phase 1 trials is to establish a safe and reliable recommended dose and schedule for upcoming phase 2 and 3 testing. In the era of molecularly targeted therapy, phase 1 trials have been conducted not only to achieve this primary goal but also to identify specific target patient populations and produce preliminary biomarker evidence of targeted therapy [11]. The most amazing example was the anaplastic lymphoma kinase (ALK) story, in which the oral c-mesenchymal-epithelial transition (c-MET) and ALK inhibitor crizotinib was approved for patients with NSCLC harboring the echinoderm microtubule-associated protein-like 4 (*EML4*)-*ALK* rearrangement by the United States (US) Food and Drug Administration (FDA) in August 2011 and by the European Medicine Agency in Autumn 2012; this occurred just after an expansion of a phase 1 trial was successfully published in 2010 [12], although the *EML4*-*ALK* rearrangement in NSCLC was discovered in 2007 [13]. Other examples of phase 1 trials that have successfully been enriched for specific target patient populations include the hedgehog inhibitor GDC-0449 in patients with advanced basal cell carcinoma [14, 15] and the v-Raf murine sarcoma viral oncogene homolog B (BRAF) inhibitor PLX4032 in patients with malignant melanoma harboring V600E *BRAF* mutations [16]. Additionally, phase 1 trials enrolling patients based on genomic analyses have a higher probability of showing efficacy than those with patients who are not genetically selected [17]. Globally, several institutes have applied next-generation sequencing techniques to the phase 1 setting for personalized oncology [18].

In the background of the rapidly evolving clinical trial system mentioned above, Dr. Jin-Ji Yang, an experienced investigator focusing on lung cancer at Guangdong Lung Cancer Institute (GLCI) in China, participated in a 4-month intensive training (September to December 2014) in the US in early-phase oncology trials sponsored by the Hengrui-US Chinese Anti-Cancer Association (USCACA) Scholarship for Early Phase Oncology Drug Development. By this chance, we performed a cross-sectional survey of early-phase oncology trials, particularly phase 1 studies of lung cancer, between the US and China.

## Materials and methods

### Brief introduction of cancer institutes visited by Dr. Yang in the US

Dr. Yang was an observer at the University of Wisconsin Carbone Cancer Center (UWCCC) in September and the University of Texas MD Anderson Cancer Center (MDACC) in December, 2014. He was a visiting fellow at the Cancer Therapy Evaluation Program at the National Cancer Institute (CTEP/NCI) in October and November, 2014. The GLCI is the largest leading lung cancer research facility in China [19]. Meanwhile, the MDACC and UWCCC are top cancer institutes in the US, particularly for high-quality early-phase oncology trials. A cross-sectional survey of early-phase trials for lung cancer was conducted between the UWCCC and GLCI in September, as well as between the MDACC and GLCI in December. Meanwhile, in addition to the MDACC, descriptive analyses of early-phase oncology trials were performed at both the CTEP/NCI and CTONG.

### Working system of lung cancer at the GLCI, MDACC, and UWCCC

The infrastructure and protocols underlying routine clinical practice and clinical trials for lung cancer were compared among the GLCI, MDACC, and UWCCC, including the names of departments, lung cancer doctors, multidisciplinary conferences, multidisciplinary clinics, grand rounds, molecular tumor boards, lung cancer working group meetings, regular academic discussions, and protocol review and monitoring committee (PRMC) meetings.

### Ongoing clinical trials for lung cancer at the GLCI, MDACC, and UWCCC

An ongoing clinical trial for lung cancer was defined as a trial specifically focusing on lung cancer and not including other solid tumors or malignancies that had been approved by the Institutional Review Board (IRB)/Independent Ethics Committee (IEC) and was open for recruiting subjects. Ongoing trials for lung cancer were classified into four types: phase 1, phase 2, phase 3, and others. Phase 1 included pure phase 1 and phase 1/2 trials. Phase 2 comprised pure phase 2 and phase 2/3 trials. Observational studies, surveys, laboratory analyses, and unclassified trials were defined as “others”. Phase 4 trials were excluded.

### Ongoing early-phase oncology trials at the MDACC

The Department of Investigational Cancer Therapeutics (ICT) focuses solely on early-phase oncology trials at the MDACC, mainly including phase 1 and 2 trials, which accounted for approximately 70 and 30% of all trials, respectively. Although Dr. Yang visited this department in December 2014, data were available on early-phase oncology trials for the period of October 2014.

### Early-phase oncology trials reviewed by the Investigational Drug Branch (IDB) at the CTEP/NCI

Early-phase oncology trials included phase 1, phase 1/2, phase 2, and “pilot” studies reviewed by the IDB in October and November 2014. A pilot study is an early trial that does not fit in as a traditional phase 1 or 2 trial. Those trials often incorporate biomarker assays that were categorized into 3 types: integral, integrated, or exploratory. For an integral type assay, the result is required for patient enrollment, or the assay is required to assess a primary objective. For an integrated type assay, the result addresses a study hypothesis or is measured to support an objective. Notably, some assays proposed in secondary objectives may actually be exploratory. In making this distinction, it may be helpful to consider how likely it is that the assay might be required as an integral marker in the next trial. An exploratory type assay is neither integral nor integrated. Data for such a biomarker assay may help to generate a new therapy hypothesis that might be tested in future clinical trials, but the assay is optional in the current study.

For the purpose of this article, preclinical data may be considered “robust” or “non-robust.” Robust preclinical data must include all the following: (1) in vitro anti-tumor activity of at least two different cell lines; (2) in vivo anti-tumor efficacy of at least two tumor types; and (3) evidence of the effect on the drug target in in vivo tumor models at doses that are plausibly achievable in humans [20]. Otherwise, the data are considered non-robust.

Proposals for early clinical trials to be sponsored by the NCI were reviewed by a panel of senior investigators at the IDB-CTEP/NCI. The outcome of the review was either “approved” or “disapproved.”

### Clinical trials for lung cancer launched by the CTONG

Since 2008, all CTONG trials for lung cancer were categorized as (neo)adjuvant epidermal growth factor receptor tyrosine kinase inhibitors (EGFR TKIs), intercalating/maintenance EGFR TKIs, first-line, second/third-line, and observational, in addition to the classifications of phases mentioned above.

### Outcome assessment

The percentage of phase 1 trials for lung cancer was compared among the GLCI, MDACC, and UWCCC. General descriptive analyses were performed for the working system of lung cancer teams among the GLCI, MDACC, and UWCCC, for the early-phase oncology trials at the MDACC and CTEP/NCI, and for CTONG lung cancer trials.

### Statistical analysis

The percentage of phase 1 trials for lung cancer and the approved rate of early-phase oncology trials were analyzed by the *χ*^2^ test (two-sided). The difference was considered significant at *P* < 0.05. Statistical computations were performed with SPSS version 13.0 (SPSS Inc., Chicago, IL, USA).

## Results

### Description of the working system of lung cancer teams at the GLCI, MDACC, and UWCCC

The infrastructure and protocols of routine clinical practice and clinical trials for lung cancer among the 3 institutes are summarized in Table 1. In general, the running mechanism was approximately the same, although the UWCCC appeared to have a relatively complete system. However, the lung cancer team, named the Department of Pulmonary Oncology, comprised medical, surgical, and radiation oncologists at the GLCI, whereas only medical oncologists comprised the Department of Thoracic Head and Neck Medical Oncology at both the MDACC and UWCCC. The latter two institutes held regular PRMC meetings. At the GLCI, such meetings are replaced by a series of monthly meetings for academic discussions.

**Table 1:** Comparison of the working system for lung cancer trials among 3 institutes

Item	GLCI	MDACC	UWCCC
Department	Pulmonary Oncology	Thoracic Head and Neck Medical Oncology	Thoracic Head and Neck Medical Oncology
Physicians	Medical, surgical, and radiation oncologists	Medical oncologists	Medical oncologists
Multidisciplinary clinic	No	No	Yes
Multidisciplinary conference	Yes	Yes	Yes
Grand rounds^a^	No	Yes	Yes
Working group meeting for trials	Every 2–3 weeks	Weekly	Weekly
PRMC meeting	No	Yes, monthly	Yes, biweekly
Molecular tumor board or discussion and strategies for incorporation of molecular characteristics	No	No	Yes
Academic discussion about translational medicine of lung cancer	Monthly	No	No
Clinical trials in September 2014		Not available	
Phase 1	3		8
Phase 2	4		8
Phase 3	4		4
Others	7		3
Clinical trials in December 2014			Not available
Phase 1	5	28	
Phase 2	5	15	
Phase 3	4	3	
Others	7	1	

*GLCI* Guangdong Lung Cancer Institute, *MDACC* MD Anderson Cancer Center, *UWCCC* University of Wisconsin Carbone Cancer Center, *PRMC* protocol review and monitoring committee.

^a^Grand Round refers to an academic lecture formally given by a specialist, expert, professor, scientist, or occasionally, a PhD student who is within a hospital/institute/university or from outside.

### Comparing the percentage of phase 1 trials for lung cancer among the GLCI, MDACC, and UWCCC

The GLCI had a significantly lower percentage of phase 1 trials than the MDACC in December 2014 (23.8% [5/21] vs. 59.8% [28/47], *χ*^2^ = 7.433, *P* = 0.006), but there was no significant difference between the GLCI and UWCCC in September 2014 (16.7% [3/18] vs. 34.8% [8/23], *χ*^2^ = 0.891, *P* = 0.345) (Table 1).

### Description of ongoing early-phase oncology trials at the MDACC

**Figure 1:**
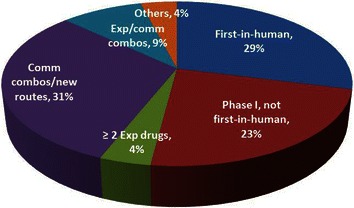
Early-phase oncology trials at the Department of Investigational Cancer Therapeutics at MD Anderson Cancer Center. *Comm* commercial, *Exp* experimental.

Data on early-phase oncology trials at the MDACC in October 2014 are shown in Figure 1. Among 149 ongoing early-phase trials, first-in-human studies accounted for 29%, and all phase 1 trials that were not first-in-human studies accounted for 23%. There were no overlapping trials between the Department of ICT and the Department of Thoracic Head and Neck Medical Oncology.

### The approval rate of early-phase oncology trials with and without robust preclinical data at the IDB-CTEP/NCI

**Figure 2:**
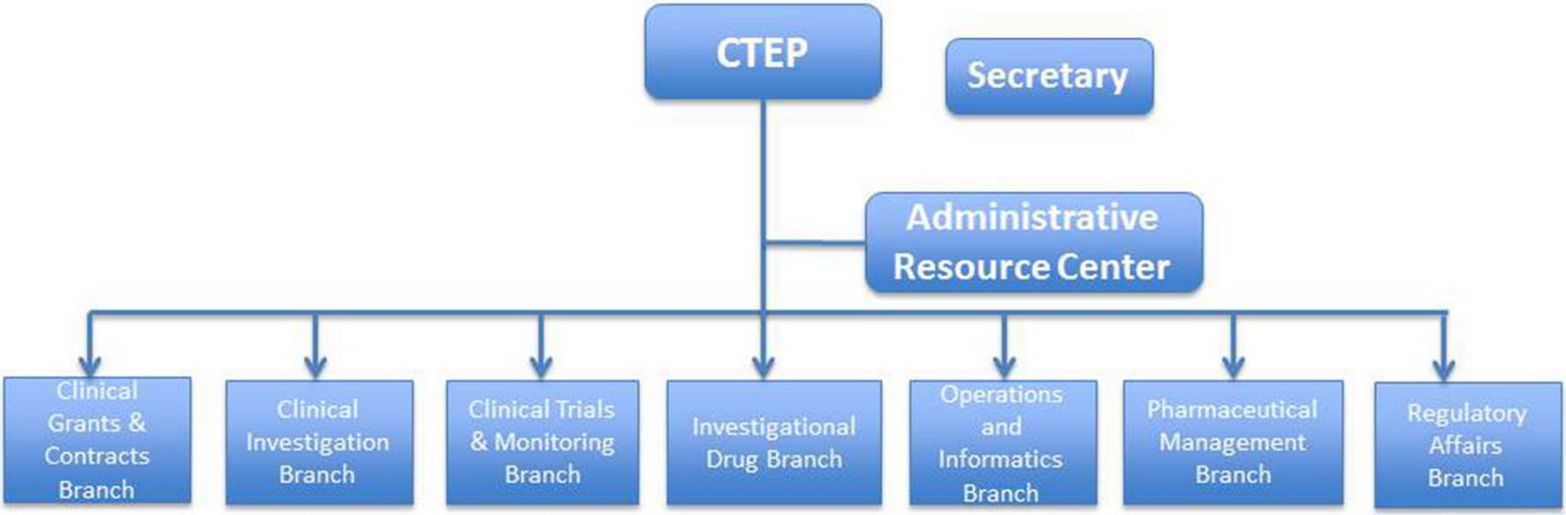
Branches of the Cancer Therapy Evaluation Program (CTEP) at the National Cancer Institute (NCI) in the United States.

The CTEP/NCI organizational chart is shown in Figure 2. Commonly observed solid tumors and hematologic malignancies were included in reviewed early-phase oncology trials. All investigational drugs and combinations had well defined targets, and biomarker identification was required in all trials. The approval rate of early-phase trials with robust preclinical data was generally higher than that of those without robust preclinical data.

### General description of clinical trials for lung cancer launched by the CTONG

**Figure 3:**
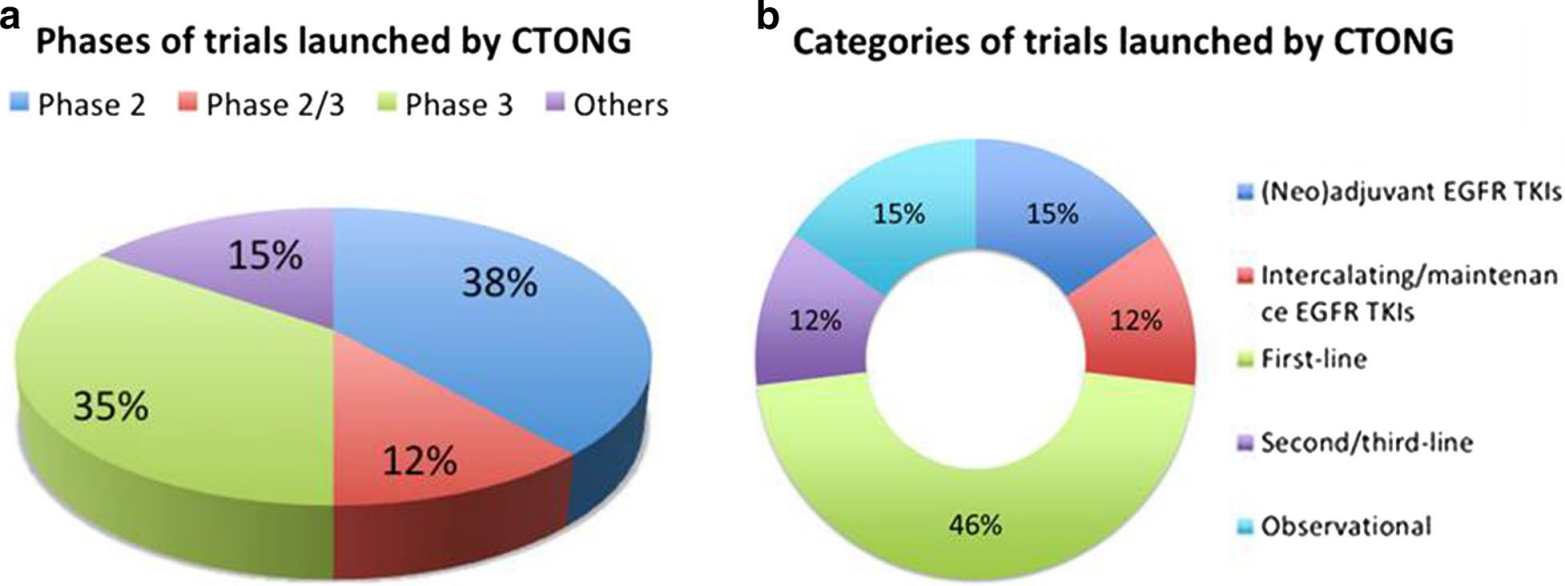
Distributions of trials for lung cancer initiated by the Chinese Thoracic Oncology Group (CTONG). **a** by phases of trials, **b** by treatments. *EGFR TKIs* Epidermal growth factor receptor tyrosine kinase inhibitors.

The CTONG was established in 2007 and launched its first trial for lung cancer in 2008. A total of 26 clinical trials for lung cancer have been initiated by the CTONG up until December 2014. Among them, phase 2, 2/3, 3, and others accounted for 38% (10/26), 12% (3/26), 35% (9/26), and 15% (4/26), respectively (Figure 3a). However, no single phase 1 trials were conducted. (Neo)adjuvant EGFR TKIs, intercalating/maintenance EGFR TKIs, first-line, second/third-line, and observational trials accounted for 15% (4/26), 12% (3/26), 46% (12/26), 12% (3/26), and 15% (4/26), respectively (Figure 3b).

## Discussion

To the best of our knowledge, this is the first cross-sectional survey of early-phase trials for lung cancer between China and the US. A significantly higher percentage of phase 1 lung cancer trials was found in the US than in China. Moreover, the early-phase oncology trials were highly strengthened at both the NCI and cancer centers in the US.

In general, the GLCI, MDACC, and UWCCC share a similar working system of lung cancer teams with multidisciplinary modalities for treating lung cancer patients. Globally, multidisciplinary teams (MDTs) have resulted in better clinical care outcomes for cancer patients, with evidence of prolonged survival among patients with a variety of solid tumors, including lung cancer [21]. Medical, surgical, and radiation oncologists comprise the Department of Pulmonary Oncology at the GLCI, resulting in more coordinated and efficient MDTs. However, no PRMC meetings occurred at the GLCI. PRMC meetings play an important role in clinical trial development and regulation [22]. It is strongly recommended that PRMC meetings be held at the GLCI as well as at large cancer institutes in China.

In December 2014, the GLCI had a significantly lower percentage of phase 1 lung cancer trials than the MDACC, although the percentage is similar to that at the UWCCC in September 2014. No phase 1 lung cancer trials were ever launched at the CTONG. Moreover, an entire department of the ICT was dedicated to early-phase oncology trials at the MDACC, which was thought to be the first phase 1 clinical trial program in the world. Over the last 5 years, the MDACC ICT conducted phase 1 first-in-human trials for 4 novel targeted agents approved by the US FDA: dabrafenib (GSK2118436) [23], trametinib (GSK1120212) [24], cabozantinib (XL184) [25], and siltuximab (CNTO328) [26]. This showcases the MDACC as the pioneer of innovative anti-cancer drug development not only in the US but also worldwide. In the era of molecularly targeted therapy, phase 1 trials based on biomarkers and cancer genomics have played a significant role in drug development and are critical to the translation of preclinical success to the practice of oncology [11–18]. To keep pace with the innovation of anti-cancer drugs globally, more early-phase, particularly phase 1, trials should be encouraged at the GLCI, CTONG, and other caner institutes in China.

As observed in the CTEP/NCI review process of early clinical trial proposals, robust preclinical data generally provide strong rationale and are considered to be of high priority for clinical evaluation. It is challenging to make appropriate decisions regarding moving new cancer agents from late preclinical development into phase 1 and from phase 1 into phase 2 trials. Among preclinical data, the extent of in vivo anti-tumor efficacy required to support the clinical development of innovative drugs remains controversial [20]. Importantly, the availability of biomarkers and biomarker assays is critical to consider in planning the approach to phase 1 clinical trials [11]. Most of the trials reviewed by the IDB have at least some degree of biomarker involvement, which also requires stringent reviews with regard to the roles of biomarkers in the trial and the analytical performances of the biomarker assays. In China, few innovative targeted anti-cancer agents for lung cancer have been successfully developed, except for icotinib, which is an EGFR TKI approved for advanced NSCLC by the China FDA (CFDA) [8]. Therefore, robust preclinical data and appropriate biomarker assays should be prioritized in the design of phase 1 trials.

**Figure 4:**
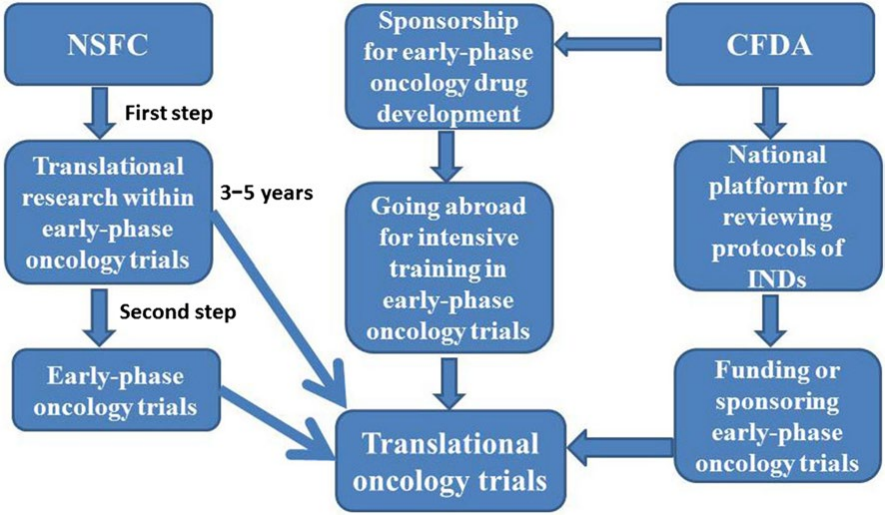
A recommendation that translational oncology trials should be facilitated by the National Natural Science Funding of China (NSFC) and China Food and Drug Administration (CFDA). *INDs* investigational new drugs.

The mission of the CTEP is to improve the lives of cancer patients by finding better ways to treat, control, and cure cancer. The CTEP accomplishes this mission by funding an extensive national program of cancer research and by sponsoring clinical trials to evaluate new anti-cancer agents, with a particular emphasis on translational research to elucidate molecular targets and the mechanisms of drug effects. As far as we know, no institution similar to the CTEP exists in China. Without government funding or sponsorship, some promising early-phase oncology trials lost the opportunity to be further academically developed. For example, the CTONG0806 study, a multi-center phase 2 trial, was initiated to explore the efficacy and tolerability of pemetrexed versus gefitinib as a second-line treatment in patients with advanced non-squamous NSCLC harboring wild-type *EGFR* [27]. The lack of funding or sponsorship led to slower accrual and much more delayed publication of CTONG0806 than two other similar trials [28, 29]. Thus, we suggest that early-phase oncology trials should be funded or sponsored by the National Natural Science Funding of China (NSFC) and CFDA to facilitate the development of innovative anti-cancer agents in China (Figure 4).

Several limitations exist in this cross-sectional survey. As an observer or visiting fellow, Dr. Yang was not authorized to obtain sufficient data directly from the UWCCC, CTEP/NCI, and MDACC, all of which withheld some data due to academic confidentiality. Therefore, the data for analysis were incomplete. Finally, none of the staff from the CTEP/NCI, MDACC, and UWCCC were directly involved in authoring this study.

In conclusion, institutes in China and the US share a similar working system of lung cancer teams for routine clinical practice and clinical trials. A higher percentage of phase 1 trials for lung cancer was observed in the US than in China. The early-phase oncology trials with robust preclinical data had a higher chance of being approved by the IDB at the CTEP/NCI. Significantly more early-phase oncology trials were conducted in the US. Early-phase oncology trials should be encouraged and funded in China.
